# Computational modeling of orthostatic intolerance for travel to Mars

**DOI:** 10.1038/s41526-022-00219-2

**Published:** 2022-08-09

**Authors:** Lex M. van Loon, Anne Steins, Klaus-Martin Schulte, Russell Gruen, Emma M. Tucker

**Affiliations:** College of Health and Medicine, Australian National University, Canberra, ACT Australia

**Keywords:** Experimental models of disease, Risk factors, Physiology

## Abstract

Astronauts in a microgravity environment will experience significant changes in their cardiopulmonary system. Up until now, there has always been the reassurance that they have real-time contact with experts on Earth. Mars crew however will have gaps in their communication of 20 min or more. In silico experiments are therefore needed to assess fitness to fly for those on future space flights to Mars. In this study, we present an open-source controlled lumped mathematical model of the cardiopulmonary system that is able simulate the short-term adaptations of key hemodynamic parameters to an active stand test after being exposed to microgravity. The presented model is capable of adequately simulating key cardiovascular hemodynamic changes—over a short time frame—during a stand test after prolonged spaceflight under different gravitational conditions and fluid loading conditions. This model can form the basis for further exploration of the ability of the human cardiovascular system to withstand long-duration space flight and life on Mars.

## Introduction

Microgravity defines an environment where gravitational forces on the human body are significantly less than those experienced on planet Earth. The fluid compartments of the human body are expectedly most prone to immediate and mid-term effects, whilst solid organ composition may adapt to altered feedback loops as most evident in the musculoskeletal system. Exposure to microgravity profoundly changes cardiovascular hemodynamics. Compared to upright posture on Earth, fluid rapidly redistributes from the bottom half to the top half of the body. Reduced venous pooling in the copious lower extremity territory is followed by a rapid contraction of plasma volume. This is primarily due to transcapillary fluid filtration into upper-body interstitial spaces, exacerbated by any reduction of fluid intake, and leads to a 10–15% reduction of the extracellular fluid volume^1–3^.

Alongside fluid maldistribution, autonomic dysfunction occurs within a few days of microgravity exposure. Whilst inapparent in space, it causes inadequate vasoconstriction and lack of responsiveness and adaptability of total peripheral resistance on standing following return to Earth^4,5^. In addition, cardiac atrophy occurs rapidly in microgravity, likely due to the reduced contractility required to maintain adequate arterial pressure^6^. Convergent lines of deconditioning cause an inability of the cardiovascular system to adapt to gravitational exposure upon a return to Earth and maintain adequate blood pressure in an upright position^4^. This is known as post (space) flight orthostatic intolerance.

Orthostatic intolerance can be due to orthostatic hypotension, neurally mediated (reflex) syncope, and postural tachycardia syndrome (POTS). Where orthostatic hypotension is defined as a sustained reduction of systolic blood pressure of at least 20 mmHg or diastolic blood pressure of 10 mmHg within 3 mins of standing or head up tilt to at least 60 degrees and POTS is defined as a sustained heart rate of >30 beats/min within 10 mins of standing or head up tilt in the absence of orthostatic hypotension. It is experienced as presyncope (symptoms of global cerebral hypoperfusion without loss of consciousness) or syncope (a transient loss of consciousness and postural tone due to global cerebral hypoperfusion followed by complete recovery) or POTS (experienced as lightheadedness, palpitations, tremulousness and weakness)^7^.

Twenty to thirty percent of astronauts returning from short duration space flights^8–10^ and ~80% of astronauts returning after long-duration space flight experience symptomatic orthostatic intolerance^11,12^, compared with only 5% of the unexposed general population under 50 years of age^5^.

Symptoms can be prevented or managed with inflight lower body negative pressure (LBNP)^13^, fluid loading, compression garments, and pharmacological therapy on re-entry into Earth’s gravitational field. In absence of a specialist ground support team, as would be the case in early missions to Mars, post-flight orthostatic intolerance after such long-duration spaceflight constitutes a significant risk to astronaut safety and mission success^13^. Management algorithms suited to all possible scenarios are needed.

Modelling the cardiovascular system engages productive cycles of insight into the underlying physiological processes^14,15^. It is a safe, cost-effective and feasible way to predict changes and responses to treatment in space travelers. The approach has been used to simulate post flight orthostatic intolerance^16,17^, short-term adaptations to low gravity and the effectiveness of the LBNP countermeasure^18^, and cardiovascular deconditioning during long-term space flight^19^. However, the effect of a prolonged (>6 months) exposure to microgravity on orthostatic intolerance has never been modelled. Neither is there any modelling available showing the response of the cardiovascular system to travelling to Mars and performing an active stand test there. The primary objective of the here presented model is to predict if humans can withstand orthostatic stress on arrival on Mars after prolonged space travel. We aimed to develop a model suitable for short- and long-duration spaceflight, simulate re-entry to Earth’s and Mars’ gravity, and validate the model using previous orthostatic stress experiments in astronauts^8,20–22^.

## Results

**Fig. 1:**
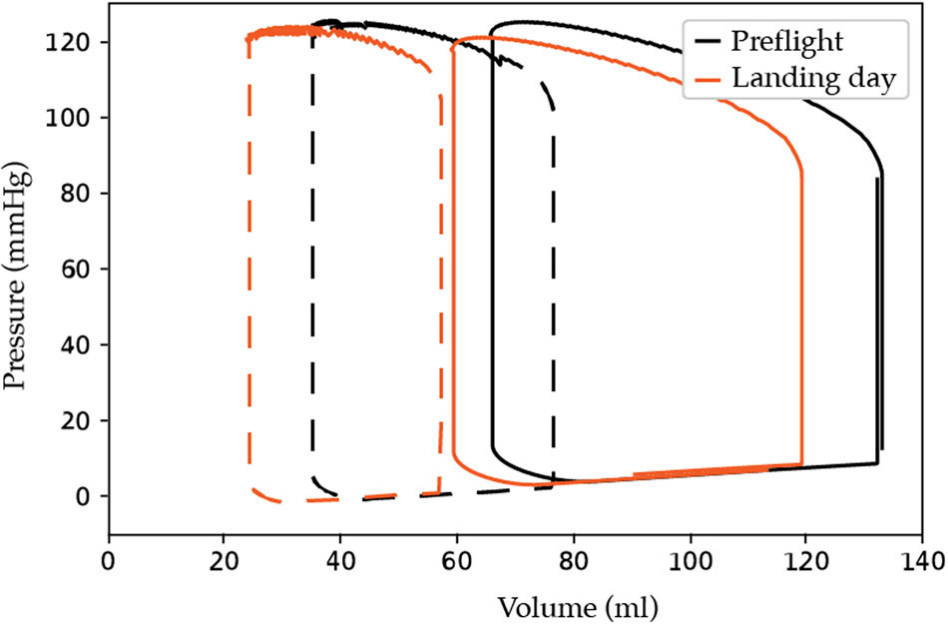
Pressure-volume loop: effect of stand test on the left ventricle before and after a short duration spaceflight. Pressure-volume loop of the left ventricle pre-flight (black) and on landing day after a short duration space flight (orange), with solid lines representing the supine position and the dashed lines after standing up.

**Fig. 2:**
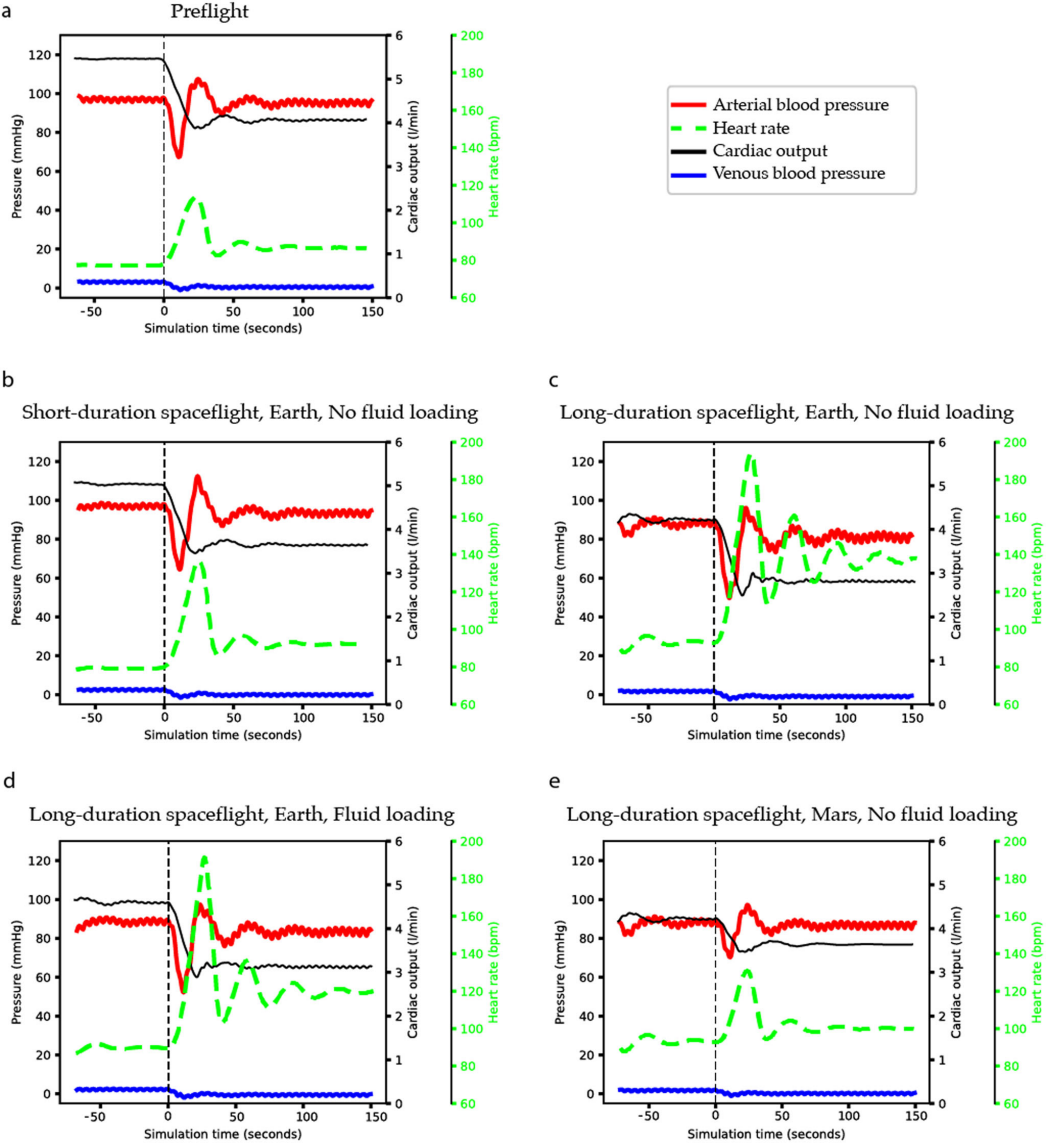
Hemodynamic responses to stand test pre-flight and on landing day under different conditions. **a** Is pre-flight, **b** Landing day after short-duration space flight (<10 days), **c** Long-duration spaceflight, no fluid loading, and Earth’s gravity, **d** Long-duration space flight, fluid loading prior to return to Earth, and Earth’s gravity, and **e** Long-duration space flight, no fluid loading, and Mars’ gravity. Line colours: dashed line = start stand test, red = mean arterial pressure, dashed green = heart rate, black = cardiac output, and blue = central venous pressure.

The key simulation results and available relevant physiological data from astronauts pre-flight and on landing day are compared in Figs. 1, 2 and Tables 1, 2. The effect of the stand test was assessed by observing the mean arterial pressure (pressure in compartment 0), the central venous pressure (pressure in compartment 15), cardiac output (flow into compartment 0), and heart rate. Results for in supine position were taken at t = −50 s and for the standing position were taken at t = 100 s.

**Table 1:** Hemodynamic changes to a stand test pre-flight and after a short-duration spaceflight.

Setting:	Pre-flight	Pre-flight	Short	Short	
	Earth	Earth	Earth	Earth	
	Simulation	Literature	Simulation	Literature	
Hemodynamic variable	Standing-supine	Standing-supine [limits]	Standing-supine	Standing-supine [limits]	Ratio
SAP (mmHg)	−10 (−11%)	−11.5 to 4	−12 (−12%)	−44.8 to −6	1.2
DAP (mmHg)	3 (0%)	0 to 9	2 (+2%)	1.5 to 7	–
MAP (mmHg)	−2 (−2%)	−2 to 5	−3 (−3%)	−30 to 2	2.2
CVP (mmHg)	−3 (−85%)	−5 to 2	−2 (−102%)	n.a.	1.2
HR (bpm)	12 (+16%)	6 to 20.6	16 (+21%)	23.5 to 41	1.3
CO (l/min)	−1.8 (−31%)	−2.5 to −1	−1.5 (−30%)	−1.5 to −1.2	0.8
SV (ml)	−32 (−40%)	−50.5 to −9	−38 (−46%)	−46 to −21.6	0.9
SVR (mmHg*l-1*min)	7 (+46%)	4.3 to 13.6	6 (+32%)	2.1 to 11.3	0.7

Values for supine were taken at t = −50 s. and for standing at t = 100 s of the simulation. All values are differences between standing and supines, except those with brackets -they indicate the percentual changes- and the ratio. The ratio shows the percentual change caused by a stand test after a short-duration space flight divided by percentual change caused by a stand test before a space flight.

*SAP* systolic arterial pressure, *DAP* diastolic arterial pressure, *MAP* mean arterial pressure, *CVP* central venous pressure, *HR* heart rate, *CO* cardiac output, *SV* stroke volume, *SVR* systemic vascular resistance., *n.a.* not available.

**Table 2:** Hemodynamic changes to an orthostatic stress test on Earth and Mars after a long-duration spaceflight.

Setting:	Long	Long	Long	Long		Long	Long	
	Earth	Earth	Earth	Earth		Mars	Mars	
	No FL	No FL	FL +	FL +		No FL	No FL	
	Simulation	Literature	Simulation	Literature		Simulation	Literature	
Hemodynamic variable	standing-supine	standing-supine	Standing-supine	standing-supine [limits]	Ratio 1	standing-supine	standing-supine [limits]	Ratio 2
SAP (mmHg)	−11 (−11%)	n.a.	−14 (−13%)	−42.4 to −5	1.2	−4 (−4%)	n.a.	0.3
DAP (mmHg)	−2 (−2%)	n.a.	0 (0%)	−21.8 to 0	0	1 (+1%)	n.a.	0
MAP (mmHg)	−6 (−7%)	n.a.	−7 (−8%)	−28.6 to −5.7	1.1	−1 (−1%)	n.a.	0.1
CVP (mmHg)	−3 (−153%)	n.a.	−3 (−124%)	n.a.	0.8	−2.0 (−92%)	n.a.	0.6
HR (bpm)	39 (+41%)	n.a.	29 (+33%)	21.6 to 45	0.8	7 (+7%)	n.a.	0.2
CO (l/min)	−1.1 (−28%)	n.a.	−1.5 (−34%)	−6.6 to −1.4	1.2	−0.4 (−10%)	n.a.	0.3
SV (ml)	−20 (−49%)	n.a.	−23 (−49%)	−63.7 to −31.9	1	−6 (−16%)	n.a.	0.3
SVR (mmHg*l-1*min)	7 (+33%)	n.a.	7 (+34%)	9.3 to 19.7	1	3 (+12%)	n.a.	0.4

Values for supine were taken at t = −50 s. and for standing at t = 100 s of the simulation. All values are differences between standing and supines, except those with brackets -they indicate the percentual changes- and the ratios. Ratio 1 shows the effect of fluid loading by dividing the percentual change of a stand test with by one without fluid loading. Ratio 2 shows the effect of performing a stand test on Mars versus on Earth by dividing the percentual change caused by a stand test performed on Mars by the same stand test when returning to Earth after a long-duration spaceflight (without fluid loading).

*SAP* systolic arterial pressure, *DAP* diastolic arterial pressure, *MAP* mean arterial pressure, *CVP* central venous pressure, *HR* heart rate, *CO* cardiac output, *SV* stroke volume, *SVR* systemic vascular resistance, *FL* fluid loading, *n.a.* not available.

### Pre-flight conditions

Representative simulated pressure-volume loops of the left ventricle during supine and standing position are shown in Fig. 1. Table 1 and Fig. 2 panel A demonstrates that all key physiological variables generated by the model are within the range of what is considered physiologically normal for our target population - i.e. well trained, healthy, adult males – for a steady state supine position as well as for the dynamic response to a stand test^11,23,24^. This pressure-volume loops show physiological correct values for systolic blood pressure (~122 mmHg), diastolic blood pressure (~81 mmHg), diastolic filling pressure (~8 mmHg), and stroke volume (~71 ml)^25^.

### Short-duration spaceflight

The hemodynamic responses to a stand test of an astronaut on landing day on Earth, after being in space for a maximum of 10 days, are shown in Table 1, Fig. 2 panel B. All values are within normal ranges of published experimental data from real astronauts performing a stand test on landing day post short-duration spaceflight^11,23^. Although the experimental data varies and include both presyncopal and nonpresyncopal subjects, the trend and the relative changes of the key hemodynamic variables simulated by our model match well with experimental data^17,19^. Table 1 shows that mean and diastolic blood pressure during supine and standing position are equal to each other and to the baseline simulation despite a significant decrease in stroke volume. The unchanged blood pressure during a stand test is in accordance with literature and can be attributed to the reflexes as indicated by the markedly increased heart rate. The significant increase in heart rate in even more pronounced on landing day compared to pre-flight conditions (ratio = 1.3) in order to counter the spaceflight induced changes to the cardiovascular system.

### Long-duration spaceflight

Table 2 and Fig. 2 show the hemodynamic responses to a stand test after a long-duration (>6 months) spaceflight with or without fluid loading, and under either Earth’s or Mars’ gravitational conditions. The simulation results from the scenario in which orthostatic stress was tested in an astronaut on landing day with fluid loading prior to returning to Earth show to be within the reported limits from experimental data.

Fluid loading in our simulations had minor positive effect on MAP (< + 2 mmHg) but did prevent a major dip in MAP during a stand test (Fig. 2). This was mainly achieved by decreasing heart rate (−5 bpm), increasing the cardiac output (+.4 l), and stroke volume (+ 6 ml) prior to the stand test.

Last, Table 2 and Fig. 2 also show results of simulating a stand test on Mars after a prolonged spaceflight. The resulting hemodynamic changes to this orthostatic stress test on Mars are less pronounced compared to when performed on Earth, even if one on Earth is preceded by fluid loading. Even more so, the stand test on Mars shows similar results to the pre-flight condition on Earth.

## Discussion

Exposure of the human body to orthostatic stress evokes prominent short-term physiological responses that aim at maintaining blood pressure. We here provide a physiological model capable of simulating these responses. Modelling outcomes adequately reflect on real-life physiology, as exemplified by model predictions of key physiological parameters following variations of parameters such as fluid loading, i.e. circulatory blood volume, or the length of exposure to microgravity. For such scenarios, model predictions are in line with real-life observations obtained in astronauts following space flight.

Simulation results confirm the observed impact of prolonged space travel on haemodynamic resilience to a stand test, and the crucial importance of fluid loading to avert adverse outcomes in terms of significant drops of mean arterial pressure and excessive tachycardia, at least in the short term. The differences between key hemodynamic variables during standing and supine position in this study are all within the physiological limits that have been reported^9,11,26^. Our model also affords a reasonable prediction of the major impact of gravity on haemodynamic outcomes. Whilst return to Earth following prolonged space travel requires adherence to fluid loading protocols, the same person will exhibit hemodynamic resilience to the much lesser gravitational challenge caused on the surface of Mars, a planet with merely 10.7% of Earth’s mass.

A stand test is a significant challenge for human physiology, as: gravity shifts half a litre of blood from the upper body to venous capacitance vessels of the lower limbs and splanchnic circulation within seconds^27^. The ability of the human body, and here the mathematical model, to maintain blood pressure while standing relies on adequate autonomic function, the key driver of peripheral resistance, on adequate blood volume and on the elastance of heart and vessels.

The pronounced orthostatic intolerance after long-duration spaceflight cannot primarily be attributed to abnormalities in the nervous system, since this reflex system is not significantly affected after a long-duration space-flight compared to shorter-duration missions. In fact, following prolonged space flight a stand test is answered by an increase of systemic vascular resistance by 71%, as opposed to merely 50% following short-term flight. Only severe impairment of this reflex system of sympathetic vasomotor activity will lead to hypotension associated with orthostatic syncope^21,28,29^. A key literature based input assumption of our modelling approach is that the autonomic function affecting orthostatic tolerance is intact after prolonged exposure to microgravity^30^. With that, our observed orthostatic intolerance would result from other factors namely less circulating fluid and structural changes to the cardiovascular system. The role of the autonomic nerves system on orthostatic intolerance would require a dedicated experimental modelling approach.

Rather, sizable inter-individual variation in baseline function and efficacy of the autonomic reflex system may explain the observed wide range of individual susceptibility to orthostatic intolerance after spaceflights. Some individuals have severe symptoms, despite fluid loading, whereas others are less affected^9^. The individual characterization of adrenergic responses to orthostatic stress may therefore be used to predict susceptible individuals before launch and who could benefit from fluid loading^20,21^. The reflex model is therefore not only quintessential for valid simulation results, but also offers opportunities to personalize the model and with that its outcome.

Mars exploration presents a number of challenges, not least as a result of its distance from Earth. NASA currently estimates a travel time of seven months^31^, a time essentially spent in microgravity. The duration of a human Mars mission is determined a long interplanetary travel time of ~7–9 months, plus the duration the crew must remain on Mars waiting for optimal planetary alignment for return travel^32^. There is limited data assessing the risk of orthostatic intolerance on exposure to Mars’ gravity^4^ and experiments that have attempted to quantify this risk using approaches based on lower body negative pressure^33^ and parabolic flight^34^ use subjects without cardiovascular deconditioning from the long-duration spaceflight of interplanetary travel.

Despite post-spaceflight orthostatic intolerance being prominent after return to Earth gravity from long-duration spaceflight, the consequences are usually minor due to mature countermeasures, ground crew support and quick recovery^11,12,26^. Therefore, risk assessment for human exploration is paramount at the Mars side, as astronauts will re-enter (albeit reduced) gravity without medical and support infrastructure, no option for medical evacuation, and transmission delays from Earth based physicians of up to twenty minutes^35^. We are not aware of any other simulation results showing that performing a stand test after a long-duration space flight in 3/8 G will not induce significant orthostatic intolerance, even in the absence of a fluid loading protocol. The simulated stand test with Mars conditions showed a drop in cardiac output of 10% despites a compensatory rise in heart rate of 7%, while the mean arterial pressure was maintained. The simulation results that the cardiovascular system is not strongly dependant on fluid loading to withstand orthostatic stress in Mars gravity is comforting, especially since the efficacy of the NASA fluid loading protocol is questionable^36,37^.

Re-exposure to Earth gravity, after being exposed to Mars and microgravity of more than 3 years, is expected to cause an extremely high rates of orthostatic intolerance from adrenergic dysfunction and significant cardiac atrophy^4^. It is not known if exposure to Mars gravity will provide mitigating/protective effects on orthostatic intolerance upon return to 1 G^4^. We speculate that given the cardiovascular stress induced by Mars gravity in our simulation is minimal, and it will be followed by the long-duration of microgravity during interplanetary travel back to Earth, any protective effect will be negligible.

Previous parabolic flight data on subjects without cardiovascular deconditioning of long duration spaceflight shows that the magnitude of blood pressure reduction and heart rate response during a stand test is dependent on gravitational loading [57] which is consistent with our results. Validation of a stand test in Mars gravity after prolonged spaceflight is not possible, but knowing that the model is capable of simulating return to Earth after a long-duration spaceflight does reassure that model responses to a change of a single model parameter (from 1 G to 3/8 G) is trustworthy.

To achieve our objective of simulating the response of the hemodynamic system to orthostatic stress after exposure to space travel in different gravitational conditions, like others^18,38^, we chose to model the hemodynamic system by a finite set of representative compartments, each of which captures the physical properties of a segment of the vascular system. In doing so, we implicitly assume that the dynamics of the system can be simulated by restricting our analysis to relatively few representative points within the cardiovascular system. Although this approach is incapable of simulating pulse wave propagation, for example, it does reproduce realistic values of beat-by-beat hemodynamic parameters^39^.

It is well established that there is a gender difference in orthostatic tolerance both for Earth bound subjects^40^ and for astronauts^41,42^ with women having significantly higher incidence of presyncope during stand tests than men. This is thought to be due to low vascular resistance^43^, decreased arterial baroreflex compensation^44^ and smaller stroke volumes^45,46^. The model presented here represents a healthy adult male astronaut and does not include any gender effects. With increasing numbers of female astronauts experiencing long-duration spaceflight, a logical extension of this work would be to include a gendered analysis which may provide further insights into cardiovascular deconditioning of spaceflight as well other disorders of orthostatic intolerance such as postural tachycardia syndrome^47^.

Current space travels, especially the long ones, have shown that exercise is key to maintain muscle strength, bone health, and cardiac performance^48^. Our model is limited in the sense that is assumes the presence of a strict exercise program during the space travel.

Last, an inherent limitation to any modelling effort is the degree of uncertainty with which numerical values can be assigned to the various parameters of the model. The origin of the parameter values we chose to assign has been provided where possible. The degree to which the model reproduces steady-state and transient hemodynamic data suggests that the present model architecture includes all the major features that contribute significantly to the transient and steady-state hemodynamic responses to orthostatic stress^15,38^.

Travelling to Mars will challenge human health and well-being. We here provide a first-layer reductionist approach assessing that it is safe to travel to Mars under the perspective of hemodynamic resilience to orthostatic stress. Future models should focus on combining modelling results of multiple organs. Especially relevant for predicting syncope in astronauts would be to extend our model with a lung and brain perfusion model. These additional organ models would also allow further insight into the effect of an inhaled gas mixture and cerebral vascular response^49^. Furthermore, adapting mathematical models of physiology from healthy subject to groups or even individuals, could enable healthcare providers to safely model and assess the impacts of space travel. It also enables providers to remotely test potential interventions to simulate their efficacy on individuals or identify adverse impacts to provide an informed and best-case treatment for a passenger or an astronaut patient. The future of medical care in space will be enabled by increasing autonomy and support from clinical decision-support systems to assess a much broader variety of prospective travellers. Healthcare providers will need greater capabilities to assess fitness to fly and for those on future space flights should be able to perform autonomous care, handling medical conditions and emergencies without immediate real-time support from Earth.

In conclusion, the presented mathematical model is capable of adequately simulating key cardiovascular hemodynamic changes - over a short time frame - during a stand test after prolonged spaceflight under different gravitational conditions and fluid loading conditions. This model can form the basis for further exploration of the ability of the human cardiovascular system to withstand long-duration space flight and life on Mars.

## Methods

### Model requirements

The main focus of this mathematical model is to simulate the response of the hemodynamic system to orthostatic stress under different gravitational conditions after exposure to space travel of short and long-duration. This response will be simulated on a short-term time scale (2–250 s). Output requirements are a number of key physiological variables that can characterize orthostatic stress and that are routinely monitored clinically, in particular pulsative arterial and venous blood pressure (both systemic and pulmonary), cardiac output, heart rate, and respiratory rate^15^. A final qualitative requirement is that this model represents the cardiorespiratory system of an averagely trained, healthy, adult male astronaut; a population for which we have explicit target data^50^.

In this paper, currently available models^18,50,51^ are extended and adapted using parameters for short- and long-duration space flights^17,19,22^. The orthostatic stress test will be simulated with Earth’s and Mars’ gravity. Model parameters will be based on literature values as much as possible. Newly introduced parameters will be chosen to target available experimental data in the best way possible. The simulation results will be validated with available orthostatic stress experiments in astronauts^8,20–22^.

### Conceptual model

**Fig. 3:**
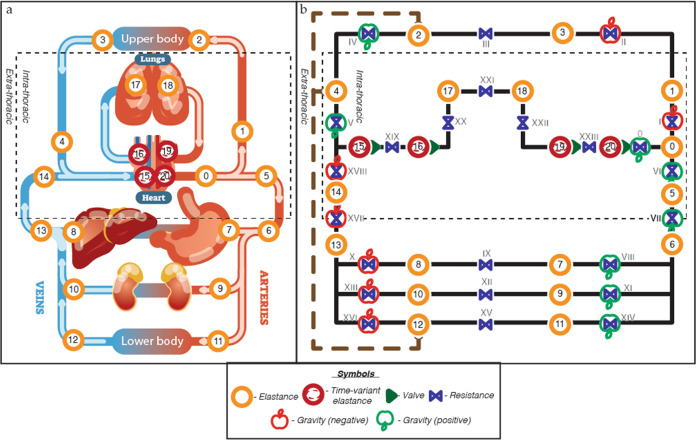
Diagram and conceptual model of the 21-compartment cardiovascular model. 0: Ascending aorta. 1: Brachiocephalic arteries. 2: Upperbody arteries. 3: Upper body veins. 4: Superior vena cava. 5: Thoracic aorta. 6: Abdominal aorta. 7: Renal arteries. 8: Renal veins. 9: Splanchnic arteries. 10: Splanchnic veins. 11: Lower body arteries. 12: Lower body veins. 13: Abdominalveins. 14: Thoracic inferior vena cava. 15: Right atrium. 16: Right ventricle. 17: Pulmonary arteries. 18: Pulmonary veins. 19: Left atrium. 20: Left ventricle. **a** Anatomic model. Dashed square indicated the intrathoracic pressure. **b** Hydraulic circuit model. The orange circles with numbers are elastic elements with a pre and post resistance in blue and annotated with Roman numbers. The cardiac compartments are illustrated with red circles and represent time-variant elastances, together with their valves (green single triangles). The dashed rectangle outlines the intrathoracic compartments, and the brown wide-dashed line with round arrowheads indicates the lymphatic flow from the lower and upper body to the super vena cava. The green and red apple indicate gravity and its direction (green = added, red = subtracted)^18,39^.

The base model used here was built upon the work of Beneken^50^, Heldt^39^ and Gerber^18^, who provide a controlled 21-compartment model of human cardiovascular system. The here presented conceptual model mimics the one described by Gerber at al.^18^ (Fig. 3).

### Mathematical model

This controlled cardiovascular response to gravity model (Fig. 3) is for the largest part described by two fundamental laws of physics. We employed the classic definition of compliance/elastance^52^ to calculate the pressure in a particular compartment based on the volume. We applied Ohm’s law to fluid mechanics^53^ to calculate flow by dividing a pressure difference by resistance, and subsequently updates the volume based on this flow. Therefore, each of the 21 compartments were characterized with an inflow/outflow resistance, elastance and unstressed volume. The volume that stretches the walls is called stressed volume and the rest is called unstressed volume^54^. The elastance governs relationship between stressed volume (total blood volume in a given compartment minus its unstressed volume) and pressure^15^. The heart compartments have both a minimum and maximum elastance in order to generate pressure. Furthermore, the elastances of the lower body venous compartments (compartment no 10, 12, and 13) were made non-linear^55^.

**Fig. 4:**
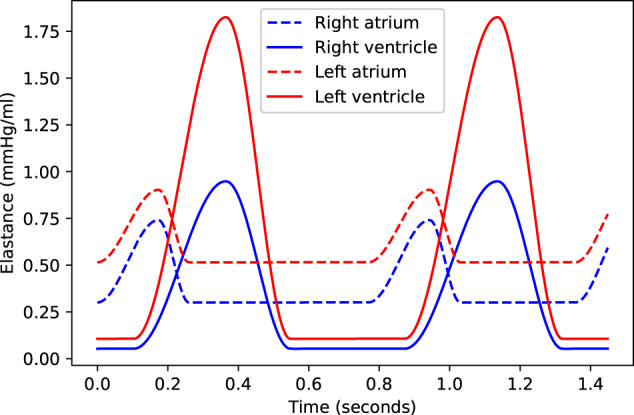
Time-varying heart elastances. Red lines indicate the left heart, and blue lines the right heart. Dashed lines indicate the atria, and solid lines the ventricles.

The heart was treated as a pressure source - together with the presence of valves - where the elastances of the four heart compartments (compartments; 15, 16, 19 and 20) switch between minimum (diastole) and maximum (systole) elastance^51^. A smooth transition between these two values was modelled by an out-of-sync sinusoidal curve^56^, with one curve representing the atria, while the other was used for the ventricles (Fig. 4). For timing of these curves, the maximum atrial elastance was placed at 0.2 s and the maximum of the ventricular elastance at 0.3 s with an offset of 0.12^57^. These timing constants were dynamically adjusted to the heart rate by multiplying them by the square root of the heart rate period^58^.

**Fig. 5:**
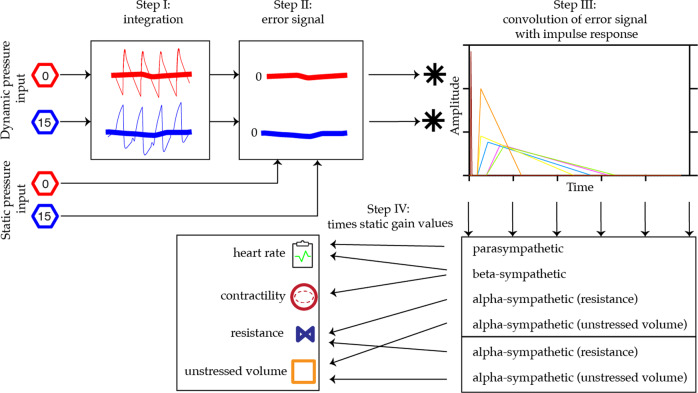
Schematic overview of the baro- and cardiopulmonary reflex control model. Step I; create a mean blood pressure, Step II; create an error signal, Step III convolute the error signal with 6 different impulse responses, and step IV; influence hemodynamic effector sides. See text section 2.3.2, ref. ^60^, and model code for more information.

The base model is controlled by arterial baroreflex (ABR) and cardiopulmonary reflex (CPR) as short-term blood pressure regulation, mimicking the sympathetic and parasympathetic nervous signals^59^. Figure 5 shows the steps that are involved in adjusting multiple cardiovascular effector sides in order to bring the two measured blood pressures (arterial and venous) close to their predefined static set-point. The reflex mechanisms are set-point controllers that aim at minimizing an error signal (see Supplementary Table 2 for reflex parameters). In short:

Step I; pressure and pulse pressure of compartment 0 (aortic arch) and pressure of compartment 15 (right atrium) were integrated over 250 data points. Step II; an error signal was created by subtracting predefined static set-points − 95 mmHg for arterial, 35 mmHg for pulse pressure and 3 mmHg for venous pressure - from this integrated signal. This error signal was subsequently scaled, as described by deBoer et al.^60^, using an inverse tangent together with scale limits of 18 and 5 for the ABR and CPR respectively. Step III; the scaled error signals were thereafter convolved with 6 different unit-area impulse response functions in order to describe the different reflex components of the autonomic nerves system. In the last step, step IV, the resulting vector from the convolution is multiplied by effector mechanism-specific static gain values to produce the 6 different effector pathways that eventually influence the heart rate, contractility, peripheral resistance, and unstressed volume^38^.

Transcapillary fluid exchange in the extremities (compartment 3 and 12) was incorporated by using Starling forces (hydrostatic and oncotic pressure) in accordance with Heldt’s and Gerber’s models^18,51,61,62^ (see Supplementary Table 1 for the parameters). This allows fluid to move from the intravascular space into the interstitial space in the upper and lower half of the body. In addition, a pathway was used to move excessive interstitial fluid via a lymphatic pump back to the superior vena cava (compartment 4). This pathway is shown with a thick brown dashed line on the left side of the conceptual model (Fig. 3).

**Fig. 6:**
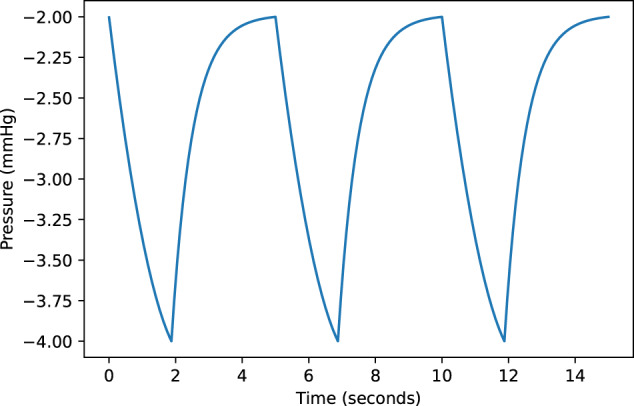
Ventilation. Simulated intra-thoracic pressure curve with a respiratory rate of 12/min.

The result of respiration induced changes in intrathoracic pressure is implemented by forcing the intrathoracic pressure on specific (intrathoracic) compartments (see Fig. 3, compartments that sits within the dashed line). The intrathoracic pressure was parameterization-based on the average profile of the respiratory muscle activity as proposed by Mecklenburgh and Mapleson^63,64^, see Fig. 6. A fixed respiratory rate of 12/min was used, with an inspiration to expiration ratio of 0.6^65^. Intrathoracic pressure is influenced by the effect of gravity on the abdomen and chest wall, and is posture dependent, therefore we used the angle of the subject with respect to the horizontal plane to adjust the intrathoracic pressure^66^. In upright postures the intrathoracic pressure decreases with increased gravity as the abdominal mass and diaphragm are pulled downwards, while this increase in gravity will increase the intrathoracic pressure in recumbent postures as the abdominal mass moves head ward^67^.

Gravitational tolerance is assessed by simulating a stand test, an active supine-to-stand task, because it is the most clinically relevant test^68^ and experimental data from astronauts post spaceflights are available for model validation^23^. The effect of standing is incorporated by either adding (green apples in Fig. 5) or subtracting (red apples in Fig. 3) a hydrostatic pressure to driving pressure of blood flow between two adjacent compartments. The hydrostatic pressures are the result of the vessel length times a gravity pressure, and was calculated using below equation.

1 $${{{\mathrm{Hydrostatic}}}}\;{{{\mathrm{pressure}}}} = \rho \; \ast \;g\; \ast \;{{{\mathrm{vessel}}}}\;{{{\mathrm{length}}}}\; \ast \;\sin (a)$$

where *ρ* is density of the blood (1060 kg/m^3), *g* is either Earth’s gravitational acceleration (9.81 m/s^2) or Mars’ (3.721 m/s^2), and the vessel length in meter (see Supplementary Table 1) is under consideration of its angle (*α*) with respect to the horizontal plane^39^. A smooth transition of gravity from zero to maximum gravity was parameterized by using a sinusoidal curve to parameterize the tilt angle alpha from 0 to 90 degrees over 5 s^18^.

Finally, muscle contraction preceding the changes in posture were incorporated by reducing the effect of the hydrostatic pressure on the intravascular pressure by a factor 2 and 3 for the legs and abdominal compartment, respectively. This reflects on the higher pressure produced by muscle contraction in the latter compartment^69^.

Oral fluid loading is often used with the intent of increasing plasma volume and maintaining mean arterial pressure during orthostatic stress^36^. The NASA fluid loading countermeasure protocol uses 15 ml/kg of body weight of water with 1 g of NaCl per 125 ml of water several hours before re-entry^36^. We assumed this protocol to be effective in the sense that it is able to increase plasma fluid by ~8%^37^. This was implemented by increasing the intravascular total blood volume with 5% at t = 0, since plasma volume is ~60% of the total blood volume^70^.

### Parameter estimation

Initial model parameters, including the reflex parameters and the resistance, elastance, unstressed volumes, vascular lengths of each cardiovascular unit were obtained from Heldt’s thesis on cardiovascular response to orthostatic stress^51^. This set of parameters describes a healthy adult male under unstressed condition in Earth’s gravity. Parameters were then adjusted to mimic both short- and long-duration space flight and different levels of gravity. No changes to the conceptual model of the cardiovascular system (i.e. vessel length or connection between compartments) when simulating the effect of microgravity. Using existing (clinical) literature, primarily following Gallo et al.^19^ and Mohammadyari et al.^17^ who did a comprehensive review of this literature for long- and short-duration space flight respectively. The rational for the long-duration setting can be found in the supplementary information of Gallo et al.’s work^19^. The detailed set of parameters can be found in Supplementary Table 1, and the rational for the adjustments in short:

First, effects of interstitial fluid shift and muscle atrophy were intrinsically taken into account in the overall setting of the spaceflight configuration, in particular by modifying the total and unstressed volumes of the relevant compartments^19^. Adjustments to the total blood volume accounts for the reduction in blood volume – this starts from the very beginning and completing with 6 weeks of space travel^71,72^ - which was reduced with 15% and 22% for short- and long-duration spaceflight respectively. Redistribution of fluid from the lower extremities to the central part of the body is relatively fast process, both legs loose up to 2 liter after 4–5 days^73,74^. Therefore, changes to the parameters accounting for the unstressed volume - which modulated volume distribution in the model - were kept equal between the short- and long-duration scenario^19^.

Second, changes to cardiac function relate to mission duration were made assuming the presence of a strict exercise program^75^. In line with Gallo et al., the changes to the right ventricle are assumed to be equal to the left ventricle. Reduction in the maximum cardiac elastances is based upon observed reduction of the contractile indexes and were therefore decreased by 27%^76,77^. Minimum left and right ventricular elastance values were about ten-fold lower compared to the maximum elastances, and therefore increased by 3%^19^. Cardiac unstressed volumes we reduced by 10%^76^.

Third, the compliances of the pulmonary compartments were increased (4% and 5% for the arterial and venous respectively)^19^. The compliance of the lower body venous system was increased with 27%. This in line with data from long-term spaceflight showing that overall venous function is changed, primarily due to muscular atrophy^78^.

Fourth, to mimic the reduction of the lower body resistances and an increase of the cerebral resistances after long-term exposure to microgravity the vessel resistance of the compartments situated above the cardiac compartment were increased with 10% whiles those below were reduced with 10%^19^.

Last, the parameters to the autonomic responses were left untouched^30^. Except for the set-points of the baroreflex, where the set-point for blood pressure was reduced (−15%) and the heart rate increased (+13%)^19,76,79–81^.

### Code implementation

To make the Python code readable and intuitive, the integration process was simplified; during each run of the model, all compartment volumes were updated by multiplying the sum of their inflow and outflow of blood with an integration step size (T = 0.001).

### Reporting summary

Further information on research design is available in the Nature Research Reporting Summary linked to this article.

## Supplementary information

Supplementary information

Reporting Summary

## Data Availability

The datasets generated and analysed during the current study are available from the corresponding author on reasonable request.

## Code availability

The model code and all model parameters are available from the corresponding author on reasonable request.
