# Supplementary material for: Computational modeling of orthostatic intolerance for travel to Mars
Source: NPJ Microgravity. 2022 Aug 9;8:34. doi: 10.1038/s41526-022-00219-2 (PMC9363491; doi:10.1038/s41526-022-00219-2)
Supplement: Supplementary file 1 — Supplementary information [file 41526_2022_219_MOESM1_ESM.pdf]

Supplementary Information

Model parameters

L.M. van Loon, A. Steins, K.-M. Schulte, R. Gruen and E.M. Tucker

Supplementary Table 1

The red numbers indicate the percentual changes for short-duration spaceflight and the purple numbers the changes for long-duration spaceflights.

#	Name	Elastance (E) [mmHg/ml]		Unstressed volume (UV) [ml]	Resistance (R) [mmHg/ml*s]		Vessel length [cm]
		Min.	Max.		In	Out	
0	Ascending aorta	3.57		21 (+4%, +4%)	0.007 (+10%)	0.003 (+10%), 0.011 (-10%)	10
1	Upper thoracic artery	7.7		5 (+4%, +4%)	0.003 (+10%)	0.014 (+10%)	4.5
2	Upper body arteries	5		16	0.014 (+10%)	4.9 (+10%)	20
3	Upper body veins	0.14		645 (+4%, +4%)	4.9 (+10%)	0.11 (+10%)	20
4	Super vena cava	0.77		16 (+4%, +4%)	0.11 (+10%)	0.028 (+10%)	4.5
5	Thoracic aorta	10		200	0.011 (-10%)	0.01 (-10%)	16
6	Abdominal aorta	10		10	0.01 (-10%)	0.1 (-10%), 0.03 (-10%), 0.09 (-10%)	14.5
7	Renal arteries	4.77		20	0.1 (-10%)	4.1 (-10%)	0
8	Renal veins	0.2		30 (-43%, -43%)	4.1 (-10%)	0.11 (-10%)	0
9	Splanchnic arteries	5		300	0.03 (-10%)	3 (-10%)	5
10	Splanchnic veins	0.015		1146 (-43%, -43%)	3 (-10%)	0.07 (-10%)	5
11	Lower body arteries	5		200	0.09 (-10%)	4.5 (-10%)	106
12	Lower body veins	0.045 (-21%)		716 (-38%, -38%)	4.5 (-10%)	0.1 (-10%)	106
13	Abdomnial veins	0.77		79 (-43%, -38%)	0.11(-10%), 0.07 (-10%), 0.1(-10%)	0.019 (-10%)	14.5
14	Inferior vena cava	2		33	0.019 (-10%)	0.008 (-10%)	6
15	Right atrium	0.74 (+3%)	0.3 (-27%)	14 (-10%, -10%)	0.008 (-10%), 0.028 (+10%)	0.006	0
16	Right ventricle	1.3 (+3%)	0.05 (-27%)	36 (-10%, -10%)	0.006	0.003 (+10%)	0
17	Pulmonary arteries	0.3 (-4%)		160 (+36%, +36%)	0.003 (+10%)	0.07 (+10%)	0
18	Pulmonary veins	0.11 (-5%)		430 (+36%, +36%)	0.07 (+10%)	0.006 (+10%)	0
19	Left atrium	0.61 (+3%)	0.5 (-27%)	11 (-10%, -10%)	0.006 (+10%)	0.01	0
20	Left ventricle	2.5 (+3%)	0.1 (-27%)	20 (-10%, -10%)	0.01	0.007 (+10%)	0

Supplementary Table 2

With red the changes for the short-duration, and in purple the relative changes for the long-duration spaceflight simulation.

Parameter	Normal	Short	Long	Unit
Respiratory rate	12			/min
Total blood volume	5250	-15%	-22%	ml
Total extravascular volume	11000	-15%	-22%	ml
	Setpoints			
HR setpoint	70		+13%	bpm
ABP setpoint	95		-15%	mmHg
PP setpoint	35			mmHg
RAP setpoint	3			mmHg
	Scaling			
ABR scale	18			dimensionless
RAP scale	5			dimensionless
	Gains			
RRs	0.012			ms/mmHg
RRp	0.009			ms/mmHg
rr_sym	0.015			PRU/mmHg
rr_para	0.09			PRU/mmHg
beta	1			ml/mmHg
alpha	1			ml/mmHg
